# Hexamoll DINCH plasticised PVC containers for the storage of platelets

**DOI:** 10.4103/0973-6247.75972

**Published:** 2011-01

**Authors:** C. S. Bhaskaran Nair, R. Vidya, P. M. Ashalatha

**Affiliations:** *R&D Center, Terumo Penpol Consultant, R&D Center, Terumo Penpol Ltd, Trivandrum, Kerala, India*; 1 *Deputy Manager, R&D Centre, Terumo Penpol Ltd, Trivandrum, Kerala, India*; 2 *Senior Executive, R&D Centre, Terumo Penpol Ltd, Trivandrum, Kerala, India*

**Keywords:** Hexamoll DINCH plasticiser, Non DEHP plasticizer, platelets, platelet storage bags

## Abstract

**Introduction:**

Containers for the storage of platelets are made using polyvinyl chloride plasticised with di, (2-ethyl hexyl) phthalate, n-butyryl, tri (n-hexyl) citrate and tri (2-ethyl hexyl) mellitate or using special poly olefins without plasticiser. Of these, the first two have disadvantages such as plasticiser leaching and impairment of platelet function. Polyolefin bags cannot be HF welded or steam sterilized. Mellitate plasticised bags can store platelets well for five days but they are not completely phthalate free.

**Research and Development:**

We have developed a new generation of containers made of PVC plasticised with the non DEHP, non aromatic plasticiser,1,2- Cyclohexanedicarboxylic acid, diisononyl ester (Hexamoll DINCH) which can store platelets without loss of function for at least six days.

**Observation:**

The present studies show that DINCH plasticised PVC bags (TPL-167) are well suited for the storage of platelet concentrates for more than five days.

**Conclusion:**

The present studies show that the PVC plasticised with the non phthalate, non aromatic, non toxic plasticiser DINCH is a viable alternative to other existing containers for the storage of platelets for more than five days.

## Introduction

Platelet concentrates have a very important role in ensuring adequate hemostasis under various clinical situations and have made possible notable advances in the medical and surgical fields. In consequence, the demand for uncontaminated viable platelets, preferably with improved storage periods is rising rapidly.

Platelets get 85% of their energy requirements by aerobic metabolism in which glucose undergoes glycolysis followed by oxidative phosphorylation of the products. Substrates such as free fatty acids and amino acids are also involved in the process. The residual 15% of the energy requirements are met by anaerobic glycolysis in which glucose is converted to lactate. The conversion of glucose to ATP by the oxidative mechanism is 18 times more efficient than anaerobic glycolysis. The carbon dioxide produced during the oxidative pathway gets converted to bicarbonate which acts as the buffer system of plasma. The containers in which platelets are stored should be carefully crafted to maintain the oxidative metabolism of the platelets. This can be achieved only if the permeability to oxygen of the containers is high. At the same time, the permeability to carbon dioxide should be high enough to permit a good part of the carbon dioxide to diffuse out but it should not be very high which would cause too much of the carbon dioxide formed to leave the container, thereby compromising the production of the bicarbonate buffer.

The first generation containers for storing RBC and platelets were made of PVC plasticised with DEHP {di-(2- ethyl hexyl) phthalate}. DEHP continues to be the plasticiser of choice for blood bags for the storage of RBCs particularly because the DEHP leached into blood plasma has a distinct protective effect on the RBC membrane which enables storage of RBC concentrates for up to 42 days. Several studies have, however, shown that leached DEHP has deleterious effect particularly for newborns, very young children and patients who require frequent blood transfusions. Hence, there is a strong move to replace DEHP with other plasticisers. DEHP-plasticised PVC containers have comparatively low permeability to oxygen and carbon dioxide and this restricts the storage period for platelets.[1–3] The leached DEHP also causes reduced aggregation responses of platelets.[4] The second generation bags overcame the permeability problem by using thinner sheets of PVC plasticised with DEHP [Teroflex XT-612 (Terumo)], and by using the plasticiser TOTM (CLX, Cutter), PL-1240 (Baxter). M/s Baxter also developed special polyolefin bags without plasticiser (PL-732). Platelet concentrates could be stored in such bags for up to five days with better preservation of function and viability.[5–11]

PVC plasticised with nbutyryl tri-(n-hexyl) citrate (BTHC) was introduced in 1989. Such bags have been shown to be acceptable for the storage of platelet concentrates and RBC.[12–15] M/s Terumo Corporation introduced a new PVC bag which was plasticised with di-(ndecyl) phthalate[16] which had very low leachability into blood plasma and was suitable for the five day storage of platelet concentrates.

The platelet storage bags in present day use have shortcomings such as phthalate contamination, compromised platelet aggregation, unpleasant odour and allergic reactions.

A new generation of PVC-based platelet storage bag [TPL 167] was developed in 2006 by M/s Terumo Penpol Ltd[17,18] in which the non DEHP, non aromatic plasticiser 1,2-cyclohexane-di carboxylic acid, diisononylester subsequently referred to as DINCH, was used. These bags were comparable to TPL’s standard TEHTM plasticised platelet storage bag (TPL-157) and other well-known platelet storage bags plasticised with TEHTM.

A second comparative assessment of TPL’s DINCH and TEHTM plasticised platelet storage bags was done at the Apollo Hospital, Chennai.

The results of these studies are presented in this paper:

## Materials and Methods

The platelet storage bags used for the study were as follows.

**Table:** Bags evaluated

Details	Plasticiser used
TPL – 157	TEHTM
TPL - 167	DINCH

These bags were part of top and bottom quadruple bag systems consisting of:

- Main bag - 450 ml
- SAGM bag - 400 ml containing 100 ml SAGM solution attached to the bottom of the main bag.
- Platelet storage bag – 400 ml (xperimental bag)
- Transfer bag – 400 ml.

### Characteristics of test bags

The characteristics of the material of the test bags are given in Table 1. The bags were also tested for physical, chemical and biological requirements for conformance with the ISO 3826 standard for blood bags.

**Table 1:** Characteristics of sheets used in this study

Property	TPL 157 TOTM	TPL 167 DINCH
Average thickness (mm)	0.4	0.4
Hardness – (shore A) ASTM D 2240	78.5	78
Tensile strength (Kg/cm^2^) ASTM D 882	170	155
Elongation at break (%) ASTM D 882	500	480
Permeability		
Oxygen (g/m^2^/24h/37°C)	1020	1070
Carbon dioxide (g/m^2^/24hr/37°C) ASTM D 1434	4850	4600
Brittle point (°C) ASTM D7028	-36	-38

### Blood collection and component separation

Blood of the same blood group was collected from four volunteer donors in SB 450 bags containing CPD and was kept without disturbance at room temperature for 1 h. The blood was pooled in a 2L capacity Terumo pooling bag using Terumo’s sterile tube sealing device (TSCD). The blood was mixed by gentle tilting. 450 ml lots of the mixed blood were then transferred into the main bags of the two QB blood bag systems (as described earlier) using TSCD. Separation of the components was done by the Buffy coat method. After a holding time of 1 h, the bags were centrifuged at 3300 rpm for 13 min at 20°C in a Hitachi Rotosilenter 630 RS centrifuge.

Three layers separated as follows:

- Top layer of platelet poor plasma
- Middle layer of Buffy coat
- RBC concentrate

### PC preparation

The RBC concentrate was transferred to the bottom bag containing 100 ml of SAGM solution and the plasma was transferred to the corresponding plasma bag. These bags were separated after sealing off the connecting tube. The Buffy coat rich in platelets remained in the main bag. It was kept suspended for 24 h at 20 - 24°C. This bag was given a soft spin at 600 rpm for 5 min at 20°C and the separated platelet concentrate was transferred to the platelet bag under test. The platelet volume was adjusted to 70 ml by the addition of plasma. The platelet bags were stored in a platelet agitator with horizontal shaking at 22°C ± 2°C. PC samples were drawn on zero, one, three, five and seven days after separation to components and the holding time of 24 h.

### Parameters studied

1. RBC count, leucocyte count and platelet count
  These were measured using the automatic blood cell counter Beckman LH 750- 5 part differential cell counter.
2. pH, pO_2_, pCO_2_, HCO_3_
  These were measured using blood gas analyzer model Bayer, Rapid Lab 248.
3. Lactate
  Measured using Hitachi – 911 UV spectrophotometer.
4. Glucose, plasma Na^+^ and K^+^
  Measured using L X 20 procedure in an automatic analyzer.
5. Aggregation

PC in which the number of platelets was adjusted to 2.5 lakhs/μl with fresh frozen autologous plasma was measured at the maximum aggregation induced by collagen at a concentration of 16 μg/ml (8 μl per test) and ADP at a concentration of 80 μmol/ml (40μl per test). Chrono -Log platelet aggregometer was used for the measurements.

## Results

Results of platelet storage evaluation studies conducted at Apollo Hospital, Chennai are given below.

### Hematological studies

The results of hematological studies are shown in Table 2. The platelet counts per experimental bag were in the range 5.1 to 9.2 × 10^10^.

**Table 2:** Hematological studies

Study parameter	Sample	Test days				
		1	2	3	5	7
Platelet count (x10^3^/μl)	TOTM					
	TPL 157-a	1063	1167	1128	1073	991
	TPL 157-b	785	881	847	786	889
	TPL 157-c	728	805	690	849	889
	TPL 157-d	863	891	853	909	775
	DINCH					
	TPL 167-a	1154	1107	1248	1052	1062
	TPL 167-b	1034	876	870	894	910
	TPL 167-c	1072	1152	1078	1150	1112
	TPL 167-d	1162	1150	1184	1140	1098
WBC count (x10^3^/μl)	TOTM					
	TPL 157-a	0.3	0.2	0.3	0.3	0.5
	TPL 157-b	0.2	0.3	0.4	0.2	0.5
	TPL 157-c	0.3	0.3	0.4	0.3	0.3
	TPL 157-d	0.3	0.2	0.3	0.3	0.3
	DINCH					
	TPL 167-a	0.5	0.5	0.5	0.4	0.4
	TPL 167-b	0.2	0.1	0.2	0.2	0.2
	TPL 167-c	0.2	0.2	0.2	0.1	0.1
	TPL 167-d	0.8	0.8	0.8	0.8	0.8
RBC count (x10^6^/μl)	TOTM					
	TPL 157-a	0.01	0.01	0.01	0.01	0.02
	TPL 157-b	0.01	0.01	0.01	0.01	0.02
	TPL 157-c	0.01	0.01	0.01	0.01	0.02
	TPL 157-d	0.01	0.01	0.01	0.01	0.02
	DINCH					
	TPL 167-a	0.02	0.02	0.02	0.02	0.02
	TPL 167-b	0.01	0.00	0.00	0.01	0.01
	TPL 167-c	0.02	0.02	0.02	0.02	0.01
	TPL 167-d	0.04	0.04	0.04	0.04	0.04

### Biochemical studies

The results of biochemical studies are shown in Table 3.

**Table 3:** Biochemical studies on stored blood

Study parameter	Sample	Test days				
		1	2	3	5	7
pH	TPL 157-a	6.95	6.99	7.04	6.94	6.76
	TPL 157-b	6.94	7.08	7.14	7.11	7.03
	TPL 157-c	6.95	7.05	7.11	7.09	7.04
	TPL 157-d	6.95	7.04	7.07	7.06	6.98
	TPL 167-a	7.15	7.25	7.29	7.27	6.95
	TPL 167-b	7.008	7.09	7.14	7.1	7.18
	TPL 167-c	7.219	7.3	7.36	7.33	7.28
	TPL 167-d	7.14	7.25	7.27	7.24	7.15
P O_2_ (mm/Hg)	TPL 157-a	37.2	82.6	114.5	134.9	124.2
	TPL 157-b	35.5	107.7	135	140.3	137.1
	TPL 157-c	40.3	91	108.9	113.2	74.4
	TPL 157-d	39.5	89.1	95	103.4	105
	TPL 167-a	105.4	121.7	125.2	142	122.4
	TPL 167-b	78.9	91	91.9	111.2	143.6
	TPL 167-c	128.4	128.2	120.9	143.4	159.6
	TPL 167-d	110.2	118.4	91.9	130.4	148.4
P CO_2_ (mm/Hg)	TPL 157-a	53.3	42	32.3	22.4	16.2
	TPL 157-b	62.4	45.7	33.3	22.4	15.2
	TPL 157-c	62.7	52	41	34.1	30.2
	TPL 157-d	59.3	48.4	42.3	33.5	31.2
	TPL 167-a	40.1	27.2	19.5	14.4	18.1
	TPL 167-b	47.9	33.8	19.1	19.1	14
	TPL 167-c	36.7	26.4	15.3	15.3	12.8
	TPL 167-d	40.4	27.4	15.1	15.1	10.7
Glucose (mg/dl)	TPL 157-a	258	275	229	197	191
	TPL 157-b	287	266	257	237	237
	TPL 157-c	295	287	274	260	273
	TPL 157-d	289	276	262	243	258
	TPL 167-a	330	317	272	252	230
	TPL 167-b	304	296	228	213	189
	TPL 167-c	388	380	409	350	330
	TPL 167-d	361	356	386	314	298
Lactate (mg/dl)	TPL 157-a	176	180	208	260	335
	TPL 157-b	134	148	169	215	271
	TPL 157-c	122	132	150	184	231
	TPL 157-d	138	151	163	206	147
	TPL 167-a	182	190	211	150	174
	TPL 167-b	225	234	268	178	202
	TPL 167-c	142	155		201	144
	TPL 167-d	175	185	197	249	174
Bicarbonate (mEq/l)	TPL 157-a	10	11	8	0	0
	TPL 157-b	12	12	9	3	4
	TPL 157-c	12	13	11	6	6
	TPL 157-d	11	13	11	5	7
	TPL 167-a	11	11	10	7	5
	TPL 167-b	10	10	6	6	5
	TPL 167-c	11	11	10	8	7
	TPL 167-d	10	9	8	6	5
Plasma K+ (mEq/l)	TPL 157-a	3.9	3.8	3.9	4	4
	TPL 157-b	3.9	3.9	3.8	4	4
	TPL 157-c	3.9	3.8	3.8	3.9	3.9
	TPL 157-d	3.8	3.8	3.8	3.9	4
	TPL 167-a	4.2	4.1	4.3	4.2	4.1
	TPL 167-b	3.9	3.8	3.9	3.9	3.8
	TPL 167-c	4.2	4.2	4.3	4.2	4.3
	TPL 167-d	4.3	4.2	4.3	4.3	4.3
Plasma Na + (mEq/l)	TPL 157-a	165	165	169	167	166
	TPL 157-b	165	165	164	165	166
	TPL 157-c	165	166	165	166	165
	TPL 157-d	164	165	166	166	167
	TPL 167-a	163	161	152	167	163
	TPL 167-b	165	165	155	165	164
	TPL 167-c	166	166	167	155	167
	TPL 167-d	168	166	166	157	168
LDH IU/L	TPL 157-a	157	180	144	155	194
	TPL 157-b	147	143	175	203	288
	TPL 157-c	145	144	155	163	192
	TPL 157-d	147	140	139	152	185
	TPL 167-a	141	165	188	176	219
	TPL 167-b	152	176	184	171	223
	TPL 167-c	123	134	127	159	154
	TPL 167-d	141	142	140	183	174

### pH, pO_2_, pCO_2_

pH was above 7.0 in all cases. The partial pressure of oxygen increased throughout preservation, while the partial pressure of carbon dioxide showed a gradual reduction. No significant differences were observed for both groups with regard to these parameters. It is clear that an oxidative atmosphere was maintained during the period of storage in both cases.

### Glucose and lactic acid

The utilization of glucose during storage was similar in samples TPL - 157 and TPL -167.

The lactate level was comparable up to the fifth day above which increase was more marked for TPL - 157.

### Bi carbonate

Decrease in bicarbonate is similar for the two samples studied.

### Plasma K^+^, Na^+^

Plasma K^+^ and Plasma Na^+^ remained fairly stable throughout the preservation.

### LDH

LDH showed a slightly increasing pattern for both the samples.

The results are similar up to five days. Beyond five days, the DINCH plasticised bags appeared to be marginally better.

### Aggregation studies

The aggregation obtained for the various samples with ADP and collagen are given in Table 4. The results show that the aggregation was maintained well for more than five days.

**Table 4:** Platelet aggregation studies on platelets stored in the test bags

Sample	Study parameter	Test days				
		1	2	3	5	7
TPL 157	Aggregation induced by ADP (%) Mean value.	-	27	10	11	5
	Aggregation induced by collagen(%) Mean value.	-	59	17	26	10
TPL 167	Aggregation induced by ADP (%) Mean value.	39	22	18	17	11
	Aggregation induced by collagen(%) Mean value.	47	52	26	15	11

## Discussions

The pH of plasma within both types of bags remained above 7.0 indicating good storage conditions. The pattern of pO_2_ change indicates adequate oxygenation of the containers. The HCO_3_ level is indicative of the presence of adequate buffer and stability consequent on the maintenance of optimum level of pO_2_ within the containers. The lactate production was within limits and comparable for the DINCH and TEHTM plasticised bags up to the fifth day, beyond which it increased significantly for the TEHTM bags. Platelet aggregation, which is an indication of platelet function, was reasonably well maintained for more than five days. It may be noted that the Buffy coat was stored for 24 h before the platelets were separated indicating one more day of storage for platelets.

The overall pattern indicates that platelet concentrates are well preserved in DINCH plasticised containers for more than five days. The results of the present study clearly show the suitability of DINCH plasticised PVC containers to preserve the function and viability of platelets in the medium concentration range. Further studies are necessary to define the range of platelet concentration which could be used, the morphology changes and the *in vivo* evaluation of platelets stored in the new type of platelet storage bag.

## Conclusions

The present studies show that the PVC plasticised with the non phthalate, non aromatic, non toxic plasticiser DINCH is a viable alternative to other existing containers for the storage of platelets for more than five days. The bags have very low odour, are non allergenic, and have low leachability into blood plasma.

## Summary

The present studies show that DINCH plasticised PVC bags (TPL-167) are well suited for the storage of platelet concentrates for more than five days.

The well-accepted platelet storage bags at present are made from special polyolefins and PVC plasticised with TEHTM or BTHC. These bags have shortcomings as pointed out earlier. DINCH plasticised PVC containers seem to be the best alternative. More studies are needed on the morphology changes and the *in vivo* evaluation of platelets stored in this new type of platelet bag.
